# Psychotherapy with somatosensory stimulation as a complementary treatment for women with endometriosis-associated pain – a qualitative study

**DOI:** 10.1186/s12906-024-04731-8

**Published:** 2024-12-26

**Authors:** Anna Limmer, Annemarie Weber, Elisabeth Olliges, Jana Kraft, Florian Beissner, Christine Preibisch, Karin Meissner

**Affiliations:** 1 Institute of Medical Psychology, Faculty of Medicine, LMU Munich, Munich, Germany; 2 Division of Health Promotion, Coburg University of Applied Sciences and Arts, Friedrich-Streib-Straße 2, 96450 Coburg, Germany; 3 Department of Psychosomatic Medicine and Psychotherapy, Klinik Barmelweid AG, Barmelweid, Switzerland; 4 Insula Institute for Integrative Therapy Research, Hannover, Germany; 5 School of Medicine, Department of Diagnostic and Interventional Neuroradiology, Technical University of Munich, Munich, Germany; 6 School of Medicine, Clinic of Neurology, Technical University of Munich, Munich, Germany; 7 School of Medicine, TUM Neuroimaging Center, Technical University of Munich, Klinikum rechts der Isar, Munich, Germany

**Keywords:** Complementary and integrative medicine (CIM), Content analysis, Endometriosis, Psychotherapy, Qualitative study, Sinosomatics

## Abstract

**Objective:**

This qualitative study aimed to explore patients’ experiences with a novel treatment approach for endometriosis-associated pain, termed ‘sinosomatics’. Specifically, it sought to understand women’s experiences of the treatment and its components, the effects of the treatment on biological, psychological, and social levels, and how the women interpreted the changes they experienced.

**Methods:**

We conducted ten semi-structured interviews with patients, who had undergone the complementary treatment for endometriosis-associated pain. These interview sessions were audio-recorded, transcribed, and analyzed using Mayring’s content analysis method with the aid of MAXQDA software.

**Results:**

Three key categories emerged: ‘treatment experience,’ ‘treatment effects,’ and ‘explanation for effects’. The treatment was described as a “turning point” in patients’ lives, offering new insights into the involvement of psychosocial factors in endometriosis-associated pain and paving the way for overcoming adverse life events. The therapy strengthened women’s empowerment, inspired hope, fostered coping strategies, and promoted personal growth. Both the holistic view of body and mind and the psychological approach to treatment led to a change in the way how women perceived the disease.

**Conclusions:**

Patients recognized the innovative combination of psychotherapy and acupuncture point stimulation as a significant advancement in managing their disease. The treatment has helped them to acquire a more holistic understanding of their bodily complaints and to cope more effectively with their symptoms. The findings highlight the importance of a patient-centered and empathetic treatment approach that empowers women to take an active role in managing their condition.

**Supplementary Information:**

The online version contains supplementary material available at 10.1186/s12906-024-04731-8.

## Introduction

Endometriosis is a benign gynecological disease characterized by the presence of endometrial tissue outside the uterine cavity [1]. There is considerable heterogeneity in the prevalence of endometriosis among the general female population, with estimates ranging from 1 to 29% [2–5]. The main symptoms of endometriosis include dysmenorrhea, dyschezia, dyspareunia, chronic pelvic pain, and infertility [6]. Patients with deep infiltrating endometriosis may present ureteric involvement, which can lead to hematuria [7]. Involvement of the vagina and the bowel can cause gastrointestinal symptoms, such as painful defecation [8].

Endometriosis impacts both sexual health and the lives of couples, primarily due to associated pain and infertility [9, 10]. Patients with endometriosis, particularly those suffering from pelvic pain, report extensive impairments in their private and professional lives [11–14]. Additionally, they often experience anxiety, depression, and a reduced health-related quality of life [13, 15–18].

Current medical approaches to treat endometriosis include surgical procedures, as well as the administration of hormonal and analgesic medications [19]. High rates of recurrence in women receiving surgical treatment [20–22], and the side effects of hormonal drugs – such as nausea, weight gain, and loss of bone mineral density (seen in therapy with GnRH-analogues) – are unsatisfactory and often limit long-term therapy [11, 20, 23, 24].

Previous research on women’s experiences with endometriosis and its psychosocial impact suggests the need for an individualized, comprehensive treatment approach [11, 18]. Young and colleagues [11] highlighted four key domains affected in patients with endometriosis: ‘life’ (impact on social, work, and sex life), ‘symptoms’ (pain and infertility), ‘medical experiences’ (diagnosis delay, treatments, and interactions with health professionals), and ‘self’ (information and knowledge, emotional well-being, and future outlook). An effective complementary therapy that encompasses many of these aspects is psychotherapy combined with somatosensory stimulation, termed ‘sinosomatics.’ This multidimensional approach integrates hypnotherapy and mindfulness techniques with somatosensory methods like acupuncture and moxibustion. Sinosomatics has been shown to significantly improve global and pelvic pain, overall well-being, and health-related quality of life in patients with painful endometriosis, with improvements persisting until follow-up at 24 months [25, 26].

In this qualitative study, we explored patients’ subjective experiences with sinosomatics. Specifically, the study aimed to understand (1) the treatment experience including the perceived relevance of different treatment components, (2) treatment effects on the biological, psychological and social health dimensions, and (3) how the women themselves explained the changes they experienced. We expected that the holistic treatment approach would foster a biopsychosocial perspective of health and disease, going along with enhanced personal responsibility for health, a deeper understanding of the body’s needs, and a heightened sense of empowerment. Furthermore, we hypothesized that, alongside acupuncture-point stimulation, both the resolution of emotional conflicts and a positive doctor-patient relationship would be perceived as essential elements of the treatment.

## Methods

### Study design

We conducted a qualitative interview study with patients who had substantial experience with sinosomatics as a complementary treatment for endometriosis and associated pain.

### Participants

Interviews were conducted with women diagnosed with painful endometriosis, all of whom had previously participated in a randomized, waitlist-controlled trial of sinosomatics and its 24-month follow-up [25, 26]. Of these, all women who had not undergone surgery or used hormonal drugs in the past 24 months were contacted via email by one of the authors (C.P.) to ask for their willingness to participate in the qualitative study. Ten participants living near Munich were selected for the interviews, including both patients with and without significant pain relief from the intervention. The endpoint for sample selection was determined to be the point of data saturation. At this stage, the information obtained from the final two interviews did not contribute any new variations to the existing categorization [27]. The qualitative study was conducted in accordance with the Declaration of Helsinki, and the study protocol was approved by the Ethics Committee of the Medical Faculty at LMU Munich (Project number 309 − 13). All participants gave written informed consent.

### Data collection

Based on the literature, we developed a semi-structured interview guide comprising nine guiding questions (Table 1). The interviews were conducted by the first author (A.L., a female medical student) between August 2013 and December 2014, as part of her doctoral dissertation project. To prepare for conducting the interviews, A.L. participated in two seminars dedicated to qualitative research methods in medicine, organized by the Institute of Sociology at LMU Munich. These seminars provided a platform for discussing a preliminary version of the interview guide. In addition, A.L. conducted two pre-test interviews with endometriosis patients experienced in sinosomatics. The transcriptions of these pre-test interviews were reviewed by the research team, which led to the refinement of the final interview guide.

**Table 1:** Interview guide

No.	Question
1.	How did you experience the Systemic Autoregulation Therapy conducted by Dr. S.?
2.	How would you describe the therapy? Please outline a standard therapy session.
3.	Did the therapy cause any changes?
4.	How do you experience your endometriosis and the pain?
5.	Please describe the connection to your body, now and prior to the therapy? (e.g. perception of body sensations, acceptance of physical deficiencies, attitude towards sexuality etc.)
6.	How do you rank the different components of therapy (according to their importance)?
7.	(If effectiveness was mentioned before:) How do you explain the effectiveness of SART?
8.	Were there any additional life events or other medical treatments since the beginning of SART that may have influenced your well-being?
9.	How would you describe the terms health and disease (now and prior to SART)?

*Note*: Systemic Autoregulation Therapy (SART) was the previous name for sinosomatics and was used at the time of the interviews

The patients were informed in advance about the objectives and scope of the study. The interviews were conducted in person, in a quiet and private setting, without the presence of other individuals. The patients were not acquainted with the interviewer. They were encouraged to speak candidly about their experiences with sinosomatics. No repeat interviews were conducted.

### Qualitative data analysis

All interviews were audio-recorded, subsequently transcribed, and managed using MAXQDA software (version 11, VERBI Software GmbH, Berlin, Germany). The transcripts were prepared by A.L. in accordance with the transcription guidelines established by Gläser & Laudel [28]. The transcription of the first interview was reviewed by the research team. Following each interview, A.L. took field notes from memory to document the interview process, the setting, the progression of the conversation, and observations from the post-interview phase.

The transcripts were analyzed by two researchers (A.L., K.M.) without prior review by the participants, according to Mayring’s content analysis [29]. The qualitative analysis primarily relied on the application of deductive categories, which were theoretically derived a priori from the interview guide. These categories and the coding guide were tested on 30% of the interview material and slightly adapted afterwards, including the introduction of additional (sub-)categories. The emerging coding tree (Additional File 1) was used to code the entire material. The results were then exported from MAXQDA to Excel for further processing, evaluation, and summarization. To ensure intersubjective comprehensibility, the qualitative analysis was supervised by two authors (A.W., J.K.) with expertise in qualitative research. We did not request feedback on the findings from the participants. In presenting the results, we selected representative quotes from the discussion transcript and translated them into English. To describe the participants’ statements, a code consisting of the letter ‘P’ followed by a number was used (P1-P10). The study was conducted adhering to the quality criteria for qualitative research [30]. The manuscript was prepared in accordance with the Consolidated Criteria for Reporting Qualitative Research (COREQ) guidelines [31, 32] (see Additional File 2).

## Results

### Interviews and participant characteristics

Out of the 27 participants who responded to the first email, 14 provided written informed consent, and 10 were contacted for interviews. All 10 interviews were completed without protocol violations and included in the data analysis. The duration of the interviews ranged from 17 to 44 min. Table 2 presents the detailed characteristics of the 10 participants at baseline (before the commencement of treatment), the number of treatment sessions they underwent, and the success of treatment.

**Table 2:** Characteristics of study participants and details regarding their sinosomatics treatment

Participant	Age (years)	Endometriosis stage (ASRM)	Maximal pelvic pain (NRS, past 3 months)	SF-12 PCS	SF-12 MCS	Number of treatments	Ongoing treatment	∆ maximal pelvic pain (NRS, past 3 months)	Perceived treatment success
P1	29	3	9	34,9	41,5	23	yes	-9,00	excellent
P2	35	4	8	47,6	47,6	15	yes	-7,00	excellent
P3	41	3	9	34,1	43	40	yes	-9,00	excellent
P4	37	2	3	58	25,8	29	no	-2,00	excellent
P5	34	2	9	47,1	24,9	25	yes	-6,00	good
P6	30	4	8	43,7	43,8	22	yes	-4,00	excellent
P7	42	3	10	36,2	50,6	20	yes	-1,00	good
P8	30	2	9	49,4	26,5	20	no	-1,00	fair
P9	40	3	5	46,5	39	34	yes	5,00	satisfactory
P10	37	2	7	37,1	51,2	36	yes	-2,00	excellent

*Abbreviations*: *ASRM*, American Society for Reproductive Medicine; *NRS*, 11-point numeric rating scale; *SF-12*, 12-Item Short-Form Health Survey; *PCS*, Physical Component Summary score; *MCS*, Mental Component Summary score

*Note*: Endometriosis stage, maximal pelvic pain, and SF-12 summary scores are based on assessments conducted before the treatment with sinosomatics. Changes (Δ) in maximal pelvic pain and perceived treatment success correspond to the 2-year follow-up of the clinical trial. Perceived treatment success was evaluated using a 5-point rating scale with the items ‘excellent’, ‘good’, ‘satisfactory’, ‘fair’, and ‘poor’

### Results of the interviews

The subcategories ‘treatment experience,’ ‘treatment effects,’ and ‘explanation for effects’ emerged as key categories and are presented in detail in the following.

#### Treatment experience

Most patients reported that they were initially “surprised” by how different the setting, the therapist, and her methods were compared to their previous experiences with doctors and clinics: *“At first*,* it was a quite new type of treatment. In the beginning*,* it was uncommon*,* but I felt good.” (P5).* They particularly emphasized the relationship with the therapist and her open, warm, and personal attitude: *“Ms. S. is a person who approaches others very*,* very openly […]. I thought to myself: ‘Oh*,* that is nice.’ It is just so unexpected. As I said*,* […] many doctors are really associated with a great distance and everything is very regulated. And she just has something cordial that meets you where you are right now.” *(P10).

In this atmosphere of trust and warmth, acupuncture was perceived as a key element: *“On the one hand*,* [the therapist’s] way of talking and focusing on the patient is very empathic. On the other hand*,* there is acupuncture*,* and there is this sudden feeling that something is happening in the body.” (P5).* Problem-oriented discussions and moxibustion were described as further important elements: *“I would say the main components were the talks*,* the acupuncture*,* and the moxibustion”* (P4). Finally, the integration of acupuncture with psychotherapeutic communication was perceived as a unique aspect of the treatment: *“So*,* she was talking to me*,* […] and while we were talking*,* or during the conversation*,* when I was saying what is kind of hurting me at the moment*,* or what happened during the week*,* or*,* I don’t know*,* what is happening at that moment*,* she then put the needles accordingly. And that has always helped me a lot“* (P3).

#### Treatment effects

Many patients reported experiencing severe pain due to endometriosis before the treatment started, sometimes leading to fainting and significant limitations in their everyday life: *“It was so bad before that I had such heavy bleeding and pain every month that I even fainted a few times. […] It is indescribable. […] These pulling pains in the abdomen are as strong as when someone stabs you in the abdomen with a knife… I was really desperate.”* (P3). Most women reported a significant improvement in endometriosis-associated symptoms, including pain and bleeding: *“The monthly bleeding […] showed a significant improvement relatively quickly – I think after three months. After six months*,* I believe*,* it became […] quite normal*,* just like for anyone else: without these extremely heavy bleedings in bursts.”* (P9).

Many patients reported that health improvements were not restricted to endometriosis, but included other symptoms such as headaches and migraine, circulatory problems, neck tension, and cystitis: *“In addition to my abdominal pain*,* I used to have very*,* very strong headaches in the past. They have gotten a lot better.”* (P7).

The treatment also improved the women’s emotional well-being: *“[I experienced the therapy] very differently from other therapies that I have done before. All in all*,* it is very comforting*,* on many levels. Not only physical*,* but also on an emotional*,* a mental level.”* (P 9). Several patients also reported feeling generally healthier and fitter, and/or less susceptible to infectious diseases since starting the complementary treatment: *“I’m much fitter now. I have much more strength*,* also for work*,* for example. [I have] a clearer head and suffer less frequent headaches and things like that”* (P6).

Prior to undergoing complementary treatment, the majority of patients experienced feelings of isolation and helplessness, coupled with a loss of hope for improvement in their condition or chances of becoming pregnant. The complementary treatment facilitated a shift in their perspective towards the disease, as well as their strategies for managing pain and limitations. This shift led many women to regain hope that their condition could be managed or potentially improved.

One patient reflected this sentiment: *“[The therapist] has the talent of giving you hope even though you gave it up years ago.”* (P7). The treatment also instilled a sense of empowerment, changing their perception from feeling helpless to taking active responsibility for their symptoms: *“I was at the mercy of endometriosis for twenty years and didn’t feel like I could do much. And in the [sinosomatics] therapy*,* I increasingly felt that I myself had a great influence on this disease.”* (P9).

Moreover, patients learned to better recognize and honor their needs and boundaries, thereby enhancing mindfulness and bodily awareness: *“You get the feeling that if you listen to your body and the pain*,* you can control it very well or work on reducing it. This kind of treatment empowers you*,* I would say*,* by training your own body awareness.”* (P10). They also acquired strategies to cope with their pain: *“When I am in pain*, I think this world is completely unfair. Why do I have this s***? I don’t want it. Why do I have it and others don’t? – Those are my first thoughts. […] And then I remember to relax,* to think about what I can do. The treatment has greatly changed this. It taught me not to see myself as a victim. To feel empowered to help myself.”* (P9).

Overall, the complementary treatment aided patients in accepting their illness and pain, integrating it as part of their lives: *“The most important thing I can say about it is that it [the therapy] changed my attitude towards endometriosis very much*,* fortunately. It is not such a drama anymore. That you suffer so insanely from it. Not just from the pain itself*,* but also from the fact that it is even there.”* (P9).

Many women reported gaining self-confidence, inner strength, and stability throughout the course of therapy: *“[…] I have the feeling that I am making personal progress. In the way you look at some things*,* or how some things are put into perspective*,* or how you can mobilize new energy for yourself.”* (P8). The treatment encouraged them to take better care of themselves, assert themselves, and set clear boundaries: *“It was quite clear that I used to pay very little attention to myself and very much to all kinds of people around me. And now I’ve learned again to pay attention to myself.”* (P6).

Participants also mentioned becoming more receptive to alternative perspectives and life choices. This openness even led some to make significant changes, such as switching jobs: *“Since I’ve been receiving treatment there*,* I’ve managed to change my employer after thirteen years. This is very difficult for someone who is very security-oriented. But I made it*,* and I’m doing much*,* much better with my new employer.”* (P1).

Thus, the treatment not only equipped women to manage their illness but also had profound effects on their lives. When asked about the impact of the therapy, P6 summed it up: *“[Interviewer] Has anything changed as a result of the therapy? [Patient] Yes. My whole life.”*

#### Explanations for the effects

A key factor attributed to the success of the treatment was the therapist’s empathy and understanding: *“And this is the first time that I feel so understood now.”* (P7). Their concerns were taken seriously: *“I think*,* what really matters*,* is that you are taken seriously […] you work on a much deeper level.”* (P1). The holistic approach was also pivotal for the patients: *“I didn’t know that doctors wanted to get to know [the patient] in depth. The usual doctor has you sitting there*,* looks at you*,* and tries to make you into a composite of different diseases. But with the [sinosomatics] therapy*,* the disease wasn’t the focus at first; it was more about the person*,* from childhood onwards with all their experiences. This understanding of the entirety of the person was actually the pleasant part.”* (P2).

The core of the treatment lay in recalling daily conflicts or past childhood experiences and addressing the resultant feelings in the present through a combination of imaginative techniques and simultaneous acupuncture: *“If something significant happened today*,* you first discuss it and attempt to recall the situation as precisely as possible: to vividly imagine how the other person appeared in that situation*,* their facial expression*,* and your own emotions. […] Most of the time*,* when you immerse yourself in the situation*,* the discomfort re-emerges immediately. You may experience abdominal pain*,* chest pressure*,* or other sensations. When this feeling arises*,* you try to create a counter-image: either by removing yourself from the situation or by protecting yourself. […] In cases of intense pain*,* you may imagine what it represents*,* such as a knife or something closely resembling the pain. You visualize removing it*,* throwing it away*,* or casting it into the sea. This process is accompanied by acupuncture*,* which is believed to alleviate the pain or the associated emotions. […] Sometimes*,* during therapy*,* pain*,* pressure*,* or negative feelings surface in other areas. In such instances*,* you continue the process until it dissipates.”* (P5). This approach helped patients in overcoming negative experiences: *“It is about overwriting a bad*,* negative feeling*,* or an experience you’ve had in your life*,* with a positive image.”* (P1) As a result, childhood issues were addressed very effectively. Furthermore, incorporating somatosensory stimulation into psychotherapeutic techniques enhanced the connection between mind and body: *“I think it is this kind of interaction between the pain you felt in the past [painful memory] and the pain you feel right now [acupuncture]. That the connection is established on a physical level*,* whereas normally you would only reach a mental level.”* (P9). In comparison to psychotherapy, sinosomatics was described as getting more to the heart of the problem: *“I think that this combination with acupuncture accelerates [the psychotherapy]. Because I had talk therapy before*,* which is much lengthier. […] The therapy [sinosomatics] gets more to the heart of it.”* (P1). One patient stated that six months of sinosomatics had been more effective than three years of psychotherapy.

Nearly all the women reported a transformative shift in their overall understanding of their disease. For instance, they grew more cognizant of how their psychological experiences impacted their illness: *“There is a reason for my pain. My body is trying to tell me something*,* whatever it may be. Perhaps it is something in the subconscious that resurfaces every month. And I believe that searching for this reason was essentially the path we followed in therapy.”* (P8). Women began to perceive their illness or pain not as an isolated punishment or fate, but rather as a response of the body to various factors in their lives: *“Suddenly*,* there is a connection and it all becomes clear. I think that is why it works so well; you find the connection and address the causes of the disease*,* rather than just tinkering with the symptoms. This is unlike other therapies I have experienced so far*,* except for SART [sinosomatics].”* (P9).

The most important facilitator for a successful therapy from the patients’ perspective was the ability to engage in the therapy: *“I could imagine that you get less from the therapy*,* if you don’t engage in the therapy*,* by saying: ‘I think that is humbug.’”* (P4). Women also emphasized that time plays a role: *“I am glad that I can continue [the therapy]. Half a year probably wouldn’t have been enough for me. […] You need time*,* or maybe it depends on what has accumulated. […] Whether there are certain situations or just one issue or if there are several problems*,* I could imagine there being differences.”* (P5).

Some study participants already had a good awareness of their own bodies before the treatment started, but sinosomatics taught them to be more attentive: *„I always tried to be aware of my body. However*,* there were times in my life when*,* despite being aware*,* I continued pushing myself*,* and the consequences were noticeable. [After therapy]*,* I learned to be more consciously aware and to act accordingly.”* (P3). One participant who did not experience sufficient pain relief reported no significant change in body attention: *“I always listened to the signals my body sent me. I always did this. And I think that did not change in a relevant manner after therapy.”* (P8).

## Discussion

The aim of the study was to learn more about the experiences of women with endometriosis-associated pain during sinosomatics treatment. The combination of problem-oriented dialogue with somatosensory stimulation techniques, such as acupuncture, was perceived as novel, different from what they had previously experienced, and effective. The treatment effects extended beyond pain relief to include general health improvements. The therapy empowered women to better cope with their condition by inspiring hope, providing coping strategies, and promoting self-care and personal growth. The therapist’s empathy, the holistic treatment of both body and mind, and a transformed understanding of the condition were highlighted as key factors in the effectiveness of sinosomatics.

A patient-centered approach, in which the patient feels taken seriously, is of particular importance in the treatment of endometriosis and has been shown to have a positive impact on patients’ health-related quality of life, particularly in terms of emotional well-being and perceived social support [33, 34]. The significant impact of endometriosis on women’s lives, their experience of illness [11–14], an often lengthy path to diagnosis [14, 35], and the stigmatization of patients with chronic pain [36, 37] may explain patients’ strong desire for respect and understanding from their treating physician. In addition, the complexity of endometriosis-associated pain may also account for the wish for an individualized treatment approach [38].

The experience of feeling understood is likely also closely related to the problem-oriented approach of sinosomatics. While standard medical treatments aim to reduce symptoms by addressing underlying biological causes, patients attributed symptom reduction during sinosomatics to specific experiences associated with autobiographic memories, negative emotions, and traumatic experiences. Recent studies have shown that women with pelvic pain respectively endometriosis are more likely to report a history of emotional, physical or sexual abuse than healthy controls [39–41]. Addressing such negative experiences is an integral part of the complementary treatment approach studied here. Therefore, an important reason for its long-lasting effectiveness may be that it acts on the intrapsychic conflicts and traumata contributing to the chronification of pain and other endometriosis symptoms.

Several patients in our study rated the combination of psychotherapy and acupuncture as more effective than the individual treatments alone, suggesting that synergistic effects may exist from combining these two methods. Given the psychosocial impact of endometriosis-related pain [11, 42] and its modulation by psychosocial factors [18, 43], a complementary treatment approach that addresses both somatic and psychological aspects appears plausible and timely.

In accordance with the literature, our results revealed that women’s concerns about the future, including symptom recurrence, fertility problems, and coping with the disease, are common [11, 44]. Sinosomatics appears to alleviate these concerns by instilling hope and empowering women to manage their condition and lead fulfilling lives in spite of it. Patients reported regaining control over their pain and adopting an active role in disease management, thus overcoming feelings of helplessness. There is consensus among chronic pain management experts on the critical importance of patient empowerment, encouraging patients to take an active role in managing their disease [45, 46]. Indicators of patient empowerment include self-efficacy, knowledge, skills, self-awareness, and attitudes [47]. Our study participants reported a shift in their attitude towards their bodies and their disease, viewing it as a meaningful bodily response to adverse life events. This perspective appeared to facilitate a deeper understanding and acceptance of their symptoms, thereby enhancing self-confidence and self-efficacy. This is in strong contrast to body-related feelings usually reported by women with endometriosis, such as a ‘sense of conflict’, of being defective, and of alienation [48]. Furthermore, sinosomatics helped women gain knowledge and skills for improved self-care, including practical self-help strategies, and taught them to pay more attention to the needs of their bodies.

### Limitations

This qualitative study has certain limitations. First, the transcription was carried out by a single researcher, potentially limiting the reliability of the findings. Second, the small sample size may restrict the external validity of the results. Nonetheless, the repetitive and consistent content across the interviews indicates that satisfactory saturation was achieved. Third, there might have been a response bias towards the medical doctor, with answers given in a socially desirable manner. This potential bias was reduced by engaging an independent researcher not involved in patient care to conduct the interviews. Fourth, restricting the study to women who had not received drug or surgical treatment during the past 24 months could have led to a selection bias toward those with more positive treatment experiences. Future studies would benefit from a detailed analysis of the characteristics distinguishing women who do and do not benefit from sinosomatics. Fifth, we interviewed women with endometriosis who consented to undergo complementary treatment with sinosomatics and the results of this study are limited to our sample. Finally, the researchers’ personal views on health and treatment might have influenced the study’s outcomes.

## Conclusions

The results provide deep insights into women’s experiences with sinosomatics as a complementary treatment for endometriosis-associated pain. Patients recognized the innovative combination of psychotherapy and acupuncture point stimulation as a significant advancement in managing their disease. The treatment has helped them to acquire a more holistic understanding of their bodily complaints and to cope more effectively with their symptoms. Women shared their experiences of feeling empowered and experiencing a decrease in feelings of helplessness. Additionally, the findings highlight the critical role of addressing emotional conflicts that may contribute to the chronic nature of pain in endometriosis. Ultimately, the results emphasize the importance of a patient-centered and empathetic approach that empowers women to take an active role in managing their condition.

## Electronic Supplementary Material

Below is the link to the electronic supplementary material.

Supplementary Material 1

Supplementary Material 2

## Data Availability

The datasets used and/or analyzed during the current study are available from the corresponding author on reasonable request.
