# Supplementary figures and images for: Psychotherapy with somatosensory stimulation as a complementary treatment for women with endometriosis-associated pain – a qualitative study
Source: BMC Complement Med Ther. 2024 Dec 26;24:426. doi: 10.1186/s12906-024-04731-8 (PMC11670555; doi:10.1186/s12906-024-04731-8)

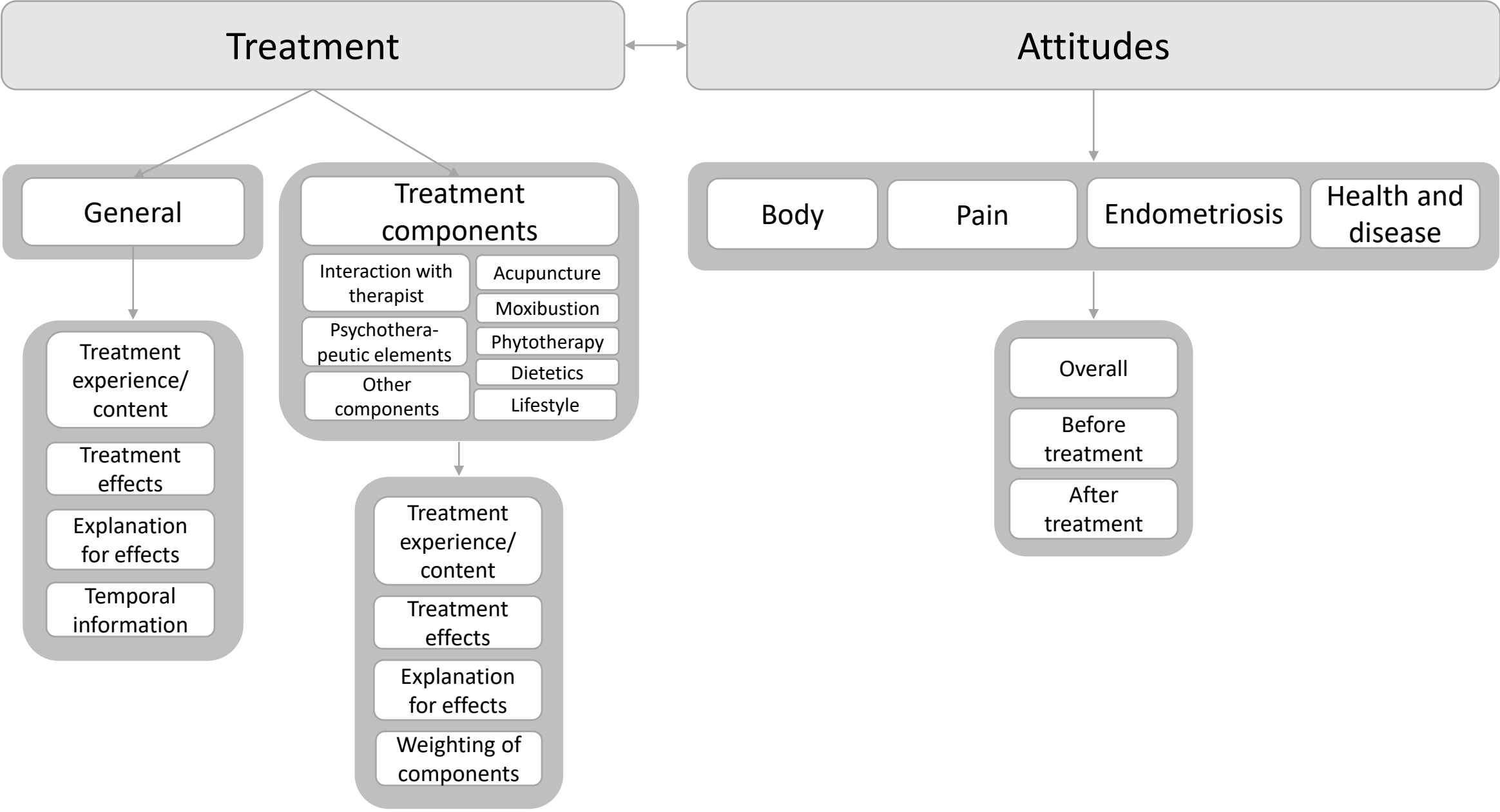

Supplement: Supplementary file 1 — Supplementary Material 1 [file 12906_2024_4731_MOESM1_ESM.pdf]
